# Profiles of Civic Engagement and Their Association with Depression, Anxiety, and Stress Among Nursing Students in Saudi Arabia

**DOI:** 10.3390/healthcare14142155

**Published:** 2026-07-17

**Authors:** Bandar S. Alharbi, Majed M. Aljabri

**Affiliations:** Community and Psychiatric Mental Health Nursing Department, College of Nursing, King Saud University, Riyadh 12375, Saudi Arabia; amajad@ksu.edu.sa

**Keywords:** civic engagement, psychological distress, depression, anxiety, nursing students, latent profile analysis

## Abstract

**Background:** Civic engagement plays an important role in promoting psychological wellbeing among university students. However, little is known about the heterogeneity of civic engagement patterns among nursing students and their association with psychological distress. We aimed to identify latent profiles of civic engagement and examine their associations with depression, anxiety, and stress. **Methods:** We performed a cross-sectional study among 255 nursing students at King Saud University. Data were collected using a structured self-administered questionnaire assessing civic engagement and psychological distress. To evaluate the psychometric properties of the civic engagement scale, we employed exploratory and confirmatory factor analyses. Latent profile analysis was used to identify distinct civic engagement profiles based on civic attitudes, civic behaviors, and community interest. We also fitted multivariable linear regression models to quantify the associations between civic engagement profiles and psychological distress outcomes. **Results:** The civic engagement scale demonstrated excellent internal consistency (Cronbach’s α = 0.93). Factor analyses supported a two-factor structure consisting of civic attitudes and civic behaviors. Latent profile analysis identified four distinct civic engagement profiles: Low engagement (16.9%), Moderate Engagement (22.0%), High civic awareness (43.1%), and Attitude Oriented (18.0%). Our findings showed significant differences across profiles in depression, anxiety, and stress scores. We observed that, compared with students in the low engagement profile, those in the Attitude Oriented profile demonstrated significantly lower depression (β = −6.63, 95% CI: −10.79 to −2.48), anxiety (β = −4.72, 95% CI: −8.87 to −0.58), and stress scores (β = −6.95, 95% CI: −10.81 to −3.09). Female students and students aged 21 to 23 years also showed significant association with lower psychological distress levels. **Conclusions:** Our findings suggested that there was distinct patterns of civic engagement among nursing students that are significantly associated with psychological distress. Higher civic engagement was associated with lower levels of depression, anxiety, and stress. This suggests that higher levels of civic engagement were associated with lower levels of psychological distress among nursing students. Strategies aimed at strengthening civic participation and community involvement may contribute to improved student mental health.

## 1. Introduction

In recent years, psychological problems among nursing students are among the major global public health issues. Several studies show high rates of depression, anxiety, and stress that substantially exceed those observed in the general student population and age-matched peers pursuing non-healthcare degrees [1,2]. According to meta-analyses and systematic reviews, the prevalence rates of such conditions were high, with depression observed in 20–54% of nursing students, anxiety occurring in 18–66% of cases, and stress affecting 46% of such individuals [3,4]. For students, the problems are multidirectional, including factors contributing to poor academic performance, clinical incompetence, and increased attrition from nurse training programs, which, ultimately, lead to substandard quality of patient care delivered by nurses once they graduate [5].

Civic engagement, on the other hand, is defined as the individual and collective actions through which citizens identify and address issues of public concern, contribute to the betterment of their communities, and actively participate in the social activities of society [6]. According to the accumulation theory, assuming socially valued roles, such as those acquired through volunteering or community leadership, confers status, identity, and a sense of purpose that buffer against psychological distress [7]. A recent scoping review found that civic engagement programs were associated with reductions in anxiety and sadness and improvements in resilience, sense of belonging, empowerment, and interpersonal skills among youth [8]. Another study further indicates that civic engagement negatively predicts depressive symptoms in young adults [9], while a systematic review of over fifty studies identified clear positive relationships between civic participation, particularly volunteerism and charitable activity, and multiple dimensions of wellbeing [10]. In a study examining these associations during the COVID-19 pandemic, civic engagement independently predicted lower levels of depression, anxiety, and burnout among young people after adjusting for sociodemographic factors [11].

Within nursing education, on the other hand, civic engagement is simultaneously recognized as a core professional value, embedded in nursing codes of ethics and accreditation standards, and as a pedagogical strategy with demonstrated benefits for professional identity formation, empathy development, and community health outcomes [12,13]. Service learning, community engagement, and student-led health initiatives have been shown to cultivate civic responsibility, deepen students’ understanding of social determinants of health, and foster the ethical and person-centered orientations essential to professional nursing practice [14]. Despite this dual relevance, there was only limited empirical evidence on the association between civic engagement and the psychological health of nursing.

This topic is particularly relevant in the Kingdom of Saudi Arabia. Under the framework of Saudi Vision 2030, volunteerism and community participation have been explicitly identified as national priorities aimed at strengthening civic responsibility and social contribution [15]. In this context, nursing students at institutions such as King Saud University represent a population that is simultaneously navigating high psychological demands associated with their academic and clinical training and operating within a societal environment that is actively promoting civic participation as a valued collective norm.

Several gaps are identified in the existing literature. Prior studies have typically adopted variable-centered approaches that treat civic engagement as a single continuous dimension, thereby obscuring the individual heterogeneity in civic engagement profiles that almost certainly exists within any given student population. The existing literature has seldom distinguished between the attitudinal and behavioral components of civic engagement, despite theoretical and empirical reasons to expect that holding civic values (attitude) and actively enacting them (behavior) may be differentially associated with psychological outcomes [10].

In this study, we aim to address the gaps using a cross-sectional survey of undergraduate nursing students at King Saud University, Riyadh, Saudi Arabia. We employed latent profile analysis to empirically identify distinct profiles of civic engagement among nursing students based on their civic attitudes, civic behaviors, and community interest. Further, we examined the sociodemographic and academic characteristics associated with profile membership. The study also assessed the independent associations between latent civic engagement profiles and each of three psychological distress outcomes, including depression, anxiety, and stress.

## 2. Method

### 2.1. Study Design and Participants

To examine the association between civic engagement profiles and psychological distress among nursing students, we performed a cross-sectional study design. Data were collected from undergraduate nursing students enrolled at the College of Nursing, King Saud University, Riyadh, Saudi Arabia. Data were collected between 16 April 2026 and 30 April 2026 using an online questionnaire administered through Google Forms. Before accessing the questionnaire, participants were provided with an electronic participant information sheet explaining the study objectives, procedures, voluntary nature of participation, confidentiality protections, and contact information for the research team. Participants were required to provide electronic informed consent by selecting an agreement option before proceeding to the survey. Only students who provided consent were able to access the questionnaire items. A self-administered online questionnaire was distributed via a QR code posted on announcement boards within the college premises, directing participants to a structured Google Form.

All undergraduate nursing students at King Saud University across all academic years (first through fourth year and internship year) were eligible. Inclusion criteria required participants to be currently enrolled nursing students aged 18 years or older. No minimum sample size restriction was applied beyond the natural enrollment pool, and all eligible students who accessed the QR code and completed the form were included in the analysis.

### 2.2. Measures

In this study, we collected data on sociodemographic variables including age (18–20, 21–23, and 24–26 years), gender (male, female), academic year (first through internship year), grade point average (GPA; below 2.5, 2.5–2.99, 3.0–3.49, and 3.5–4.0), volunteering frequency (never, rarely, occasionally, monthly, and weekly or more), and community participation (no and no plans, no but plan to, yes occasionally, and yes regularly).

Civic engagement was assessed using a 14-item scale, which included two theoretically distinct subscales: (1) Civic Attitude (8 items; for example, belief in civic responsibility, social awareness, and community values) and (2) Civic Behavior (6 items; e.g., active participation in community activities, volunteerism, and civic action). Items were rated using a 5-point Likert scale ranging from 1 (strongly disagree) to 5 (strongly agree). Subscale scores were calculated as the mean of the corresponding items, with higher scores indicating greater civic engagement. Consequently, the resulting subscale scores retained the original 1 to 5 response metric. The psychometric properties of this instrument were evaluated in the present sample; internal consistency was examined using Cronbach’s alpha, and the two-factor structure was verified through both exploratory factor analysis (EFA) and confirmatory factor analysis (CFA). Community interest was assessed using a single item asking participants to rate their interest in community engagement on a scale from 1 to 10, with higher scores indicating greater interest in community involvement. This measure was analyzed separately from the Civic Attitude and Civic Behavior subscales and was included in the latent profile analysis as an indicator of motivational engagement toward community activities.

To measure psychological distress, we used the Depression Anxiety Stress Scales–21 (DASS-21), a widely validated 21-item self-report instrument developed by Lovibond and Lovibond (1995) [16]. The DASS-21 is a short form of the original 42-item DASS and measures three negative emotional states across three subscales of seven items each: *Depression* (items 3, 5, 10, 13, 16, 17, and 21), *Anxiety* (items 2, 4, 7, 9, 15, 19, and 20), and *Stress* (items 1, 6, 8, 11, 12, 14, and 18). Participants rated each item on a four-point Likert scale ranging from 0 (*did not apply to me at all*) to 3 (*applied to me very much or most of the time*). Scores for each DASS-21 subscale were calculated by summing the responses to the corresponding seven items and multiplying the total by two, in accordance with the standard DASS-21 scoring procedure. Higher scores indicate greater severity of depression, anxiety, and stress, respectively, and permit direct comparison with the severity classifications of the original DASS-42 instrument. The DASS-21 has demonstrated robust psychometric properties in previous studies that applied it across healthcare and nursing students [17,18], and its Arabic version has been validated for use in Saudi Arabian and broader Arab populations [19].

### 2.3. Statistical Analysis

We assessed internal consistency reliability for all scales using Cronbach’s alpha coefficient, with values ≥ 0.70 considered acceptable. The factorial validity of the civic engagement scale was examined in two sequential steps. First, exploratory factor analysis (EFA) was conducted using maximum likelihood estimation with oblimin rotation to identify the latent factor structure. The Kaiser–Meyer–Olkin (KMO) measure of sampling adequacy and Bartlett’s test of sphericity were computed to confirm the suitability of the data for factor analysis. The optimal number of factors was determined by parallel analysis. Then, we performed confirmatory factor analysis (CFA) with the robust maximum likelihood estimator (MLR). Although the civic engagement items were measured using Likert-type response scales, they were treated as approximately continuous indicators. The robust maximum likelihood estimator was selected because it provides robust standard errors and model fit statistics that are less sensitive to moderate violations of multivariate normality. In addition, simulation studies have demonstrated that MLR performs adequately for ordinal indicators with five or more response categories, particularly in studies with moderate sample sizes [20]. Model fit was evaluated using the Comparative Fit Index (CFI), Tucker–Lewis Index (TLI), Root Mean Square Error of Approximation (RMSEA), and Standardized Root Mean Square Residual (SRMR), with acceptable fit indicated by CFI and TLI > 0.90, RMSEA < 0.08, and SRMR < 0.08.

To identify distinct subgroups of nursing students based on their civic engagement characteristics, we performed Latent Profile Analysis (LPA). Models with equal variances and zero covariances were estimated for one through five latent profiles. The model with the lowest information criteria and highest entropy was selected as the best-fitting solution. The three indicator variables entered into the LPA were: civic attitude score, civic behavior score, and community interest. Prior to analysis, all indicator variables were standardized (mean = 0, SD = 1) to place them on a comparable scale. Each participant was assigned to the profile for which they had the highest posterior probability, and latent profile membership was retained as a categorical variable for subsequent analyses. Model selection was based on a combination of statistical fit indices (AIC, BIC, sample size adjusted BIC, and BLRT), entropy, profile interpretability, and class size adequacy. Solutions containing very small classes (<5% of the sample) were considered less favorable because of concerns regarding profile stability and generalizability. The final model was selected based on overall statistical performance and substantive interpretability.

We conducted descriptive statistics for all sociodemographic, civic engagement, and psychological distress variables. Continuous variables are presented as mean (standard deviation; sd), and categorical variables are presented as frequency and percentage. Between-profile differences in sociodemographic and clinical characteristics were examined using one-way analysis of variance (ANOVA) for continuous variables and chi-square test for categorical variables. When the assumptions of the chi-square test were not satisfied because of sparse cell frequencies, chi-square tests with Monte Carlo simulated *p*-values based on 10,000 replicates were used. Bivariate correlations among civic engagement subscales and psychological distress outcomes were assessed using Pearson’s correlation coefficients.

To examine the independent associations between latent civic engagement profiles and each psychological distress outcome, we fitted three separate multivariable linear regression models with depression score, anxiety score, and stress score as the dependent variables. In each model, the low engagement profile served as the reference category for the civic engagement profile variable. We adjusted for potential covariates including age, gender, academic year, and GPA.

All analyses were performed using R statistical software (R version 4.2.1) [21].

## 3. Results

Table 1 presents a descriptive summary of participants’ characteristics. Among the total 255 participants, most participants were aged 21 to 23 years (57.3%), followed by those aged 18 to 20 years (29.0%), while only 13.7% were aged 24 to 26 years. Male students represented approximately two-thirds of the sample (67.5%). Several participants were in their third academic year (51.0%). Second-year and fourth-year students each represented around one-fifth of the sample, at 18.4% and 18.0%, respectively. Nearly half of the students reported a GPA between 3.5 and 4.0 (44.3%), and 36.1% had a GPA between 3.0 and 3.49. Only 3.9% of participants had a GPA below 2.5. Regarding students’ volunteering behavior, occasional volunteering was the most commonly reported frequency (35.7%), followed by rare volunteering (28.6%). About 14.5% of students reported never participating in volunteer activities, while only 8.2% engaged in volunteering on a weekly basis. About 43.9% of students reported that they were not currently participating in community activities but planned to do so in the future. Approximately one-third (30.6%) participated occasionally, whereas only 12.9% reported regular community participation.

The mean depression, anxiety, and stress scores were 12.73 (sd = 10.59), 12.64 (sd = 10.87), and 13.77 (sd = 9.81), respectively. The overall mean civic engagement score was 5.28 (sd = 1.20).

Pearson correlation coefficients among civic engagement dimensions and psychological distress outcomes are presented in Supplementary Figure S1. Civic attitude, civic behavior, and overall civic engagement scores were negatively correlated with depression, anxiety, and stress scores. Correlation coefficients ranged from weak to moderate magnitude (approximately r = −0.19 to −0.32), indicating that higher levels of civic engagement were generally associated with lower psychological distress among nursing students.

In our study, the reliability analysis demonstrated excellent internal consistency for the civic engagement scale, with a Cronbach’s alpha of 0.93 (Table 2). Similarly, the depression and anxiety subscales showed excellent reliability, each yielding an alpha coefficient of 0.90, while the stress subscale demonstrated good internal consistency with an alpha of 0.86. Thus, the study instruments had satisfactory psychometric properties that were appropriate for subsequent analyses.

Latent profile analysis was conducted to identify distinct patterns of civic engagement among nursing students. Model fit statistics for the one- through five-class solutions are presented in Table 2. Information criteria (AIC, BIC, and SABIC) decreased progressively as the number of classes increased, with the five-class solution demonstrating the lowest values and the highest entropy (0.846). In addition, the bootstrap likelihood ratio test remained statistically significant across successive model comparisons, indicating improved model fit with the addition of latent classes.

Although the five-class solution yielded the lowest AIC, BIC, and SABIC values, one latent class contained only 4.7% of participants, raising concerns regarding class stability and substantive interpretability. Consistent with recommendations for latent profile analysis, model selection considered statistical fit indices together with classification quality, class size, and theoretical interpretability. Therefore, the four-class solution was selected as the final model based on a combination of statistical fit, profile interpretability, and adequate class sizes. The selected model demonstrated good classification quality (entropy = 0.805), favorable information criteria (BIC = 1894.82; SABIC = 1837.76), and well-defined profile sizes ranging from 16.9% to 43.1% of participants.

The four identified profiles accounted for 16.9%, 22.0%, 43.1%, and 18.0% of the sample, respectively. This distribution indicates substantial heterogeneity in civic engagement patterns among nursing students. The largest subgroup consisted of students classified as having high civic awareness, representing 43.1% of the study population. The findings support the presence of distinct civic engagement profiles and provide a basis for examining their associations with depression, anxiety, and stress.

Table 3 presents the distribution of sociodemographic characteristics and psychological distress scores across the four latent civic engagement profiles identified among nursing students. Significant differences were observed across profiles for gender, academic year, GPA, volunteering frequency, and psychological distress outcomes.

We found a significant difference by gender distribution across profiles (*p* < 0.001). The High Civic Awareness profile consisted predominantly of female students (67.3%), whereas the remaining profiles were largely composed of male students. Our findings suggest potential gender differences in patterns of civic engagement and community awareness among nursing students. Academic year was also significantly associated with civic engagement profiles (*p* = 0.010). Students in the High Civic Awareness profile had the highest proportion of internship year students (16.4%). This indicates that civic awareness may increase with academic progression and clinical exposure.

Significant differences were further observed in GPA distribution across profiles (*p* = 0.005). Students in the Attitude Oriented profile demonstrated the highest academic performance, with 58.7% reporting a GPA between 3.5 and 4.0. In contrast, students in the Low Engagement profile showed a greater proportion of lower GPA categories. This means that a positive relationship between civic engagement and academic achievement.

Volunteering frequency also varied significantly between profiles (*p* = 0.043). Students in the High Civic Awareness profile most commonly reported occasional volunteering (42.7%), whereas students in the Attitude Oriented profile more frequently reported rare volunteering and lower levels of regular participation. This pattern indicates heterogeneity in the behavioral expression of civic engagement across profiles. We also observed high differences in psychological distress across the four profiles. Students in the Low Engagement and Moderate Engagement profiles demonstrated the highest mean depression, anxiety, and stress scores. In contrast, students in the High Civic Awareness profile consistently exhibited the lowest levels of psychological distress, with mean depression, anxiety, and stress scores of 9.07, 8.24, and 10.89, respectively. The Attitude Oriented profile also demonstrated lower distress levels compared with the Low and Moderate Engagement groups.

The results of exploratory factor analysis supported the multidimensional structure of the civic engagement scale among nursing students (Supplementary Table S1). The Kaiser–Meyer–Olkin value of 0.93 and the highly significant Bartlett’s test of sphericity indicated excellent sampling adequacy and suitability for factor analysis. Parallel analysis suggested a two-factor solution corresponding to civic attitudes and civic behaviors. Two Civic Attitude items (a7 and a8) showed somewhat lower loadings (0.53 and 0.57, respectively), while one Civic Behavior item (b3) exhibited moderate cross-loading on both Civic Attitude (0.38) and Civic Behavior (0.48). Nevertheless, all items exceeded commonly accepted retention thresholds and were retained because of their conceptual relevance and contribution to the overall factor structure. The two factors explained 59% of the total variance and showed a moderate positive correlation (r = 0.67), suggesting that civic attitudes and civic behaviors represent related but distinct dimensions of civic engagement. Overall model fit indices demonstrated acceptable fit, including a Tucker–Lewis Index of 0.92 and a low residual error (RMSR = 0.04), supporting the factorial validity of the civic engagement instrument.

**Figure 1:**
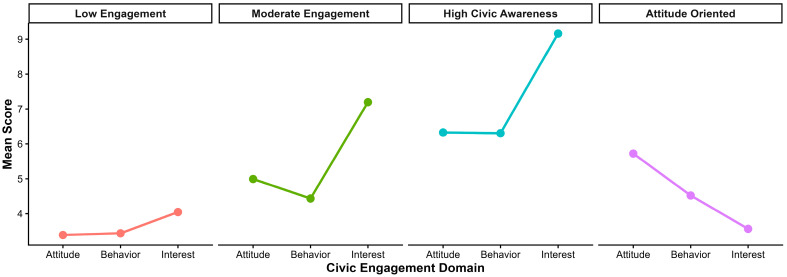
Latent civic engagement profiles among nursing students based on civic attitudes, civic behaviors, and community interest scores.

Confirmatory factor analysis further supported the two-factor structure identified in the exploratory analysis (Supplementary Table S2). All standardized factor loadings were statistically significant and ranged from 0.70 to 0.82 for civic attitude items and from 0.69 to 0.82 for civic behavior items. This indicates a strong association between observed indicators and their corresponding latent constructs. The latent correlation between civic attitudes and civic behaviors was high (r = 0.78), which indicates that there was substantial conceptual overlap while still supporting construct distinction. Model fit indices indicated acceptable overall fit (CFI = 0.921, TLI = 0.906, SRMR = 0.052). The RMSEA was 0.096 (90% CI: 0.083–0.109), indicating a somewhat elevated level of approximation error. Overall, the CFA findings supported the adequacy of the proposed two-factor measurement model. Figure 1 illustrates four distinct civic engagement profiles identified among nursing students. We observed that the Low Engagement profile demonstrated low scores across civic attitudes, civic behaviors, and community interest, which shows limited civic involvement overall. The Moderate Engagement profile showed moderate civic attitudes and behaviors accompanied by elevated community interest. This means students have a growing interest in community-related activities despite only moderate levels of active engagement. Our findings showed that the third profile, labeled high civic awareness, exhibited the highest scores across all domains, particularly for community interest. This profile reflects students with strong civic attitudes, active participation behaviors, and substantial interest in community involvement. The fourth profile was characterized by relatively positive civic attitudes but comparatively lower civic behavior and markedly lower community interest and was therefore labeled Attitude Oriented. This pattern suggests students who endorse civic values and responsibilities conceptually but exhibit lower practical participation and reduced interest in community activities.

We presented the results of the multivariable linear regression analyses examining the association between civic engagement profiles and psychological distress outcomes after adjusting for age, gender, academic year, and GPA in Table 4. The finding showed that compared with students in the Low Engagement profile, those in the High Civic Awareness profile demonstrated significantly lower depression scores (β = −4.85, 95% CI: −9.13 to −0.57). Although reductions in anxiety and stress scores were also observed in this group, the association did not reach statistically significant.

Students in the Attitude Oriented profile exhibited lower levels of psychological distress across all outcomes. Specifically, they reported significantly lower depression (β = −6.63, 95% CI: −10.79 to −2.48), anxiety (β = −4.72, 95% CI: −8.87 to −0.58), and stress scores (β = −6.95, 95% CI: −10.81 to −3.09) compared with students in the Low Engagement profile. Our findings suggest that higher civic engagement is associated with better psychological wellbeing among nursing students.

We also observed that age was associated with psychological distress. Students aged 21 to 23 years had significantly lower depression, anxiety, and stress scores compared with those aged 18 to 20 years. No significant differences were observed for students aged 24 to 26 years. Female students reported significantly lower psychological distress scores than male students across all three outcomes. Compared with males, female students had lower depression (β = −5.75, 95% CI: −9.61 to −1.89), anxiety (β = −7.61, 95% CI: −11.46 to −3.76), and stress scores (β = −6.00, 95% CI: −9.59 to −2.41). We did not find a significant association between GPA and psychological distress.

Prior to interpretation of the regression models, diagnostic assessments were performed (Supplementary Figure S2). Visual inspection of the residuals versus fitted, normal Q-Q, scale-location, and residuals versus leverage plots indicated no substantial violations of linearity, homoscedasticity, normality of residuals, or influential observations.

## 4. Discussion

In this study, we identified distinct patterns of civic engagement among nursing students and demonstrated significant associations between civic engagement profiles and psychological distress outcomes. We identified four latent civic engagement profiles, classified as Low Engagement, Moderate Engagement, High Civic Awareness, and Attitude Oriented profiles. Our findings showed that higher civic engagement was significantly associated with lower depression, anxiety, and stress scores. The findings overall suggest that civic engagement may function as an important protective factor for psychological wellbeing among nursing students.

The identification of heterogeneous civic engagement profiles in our study suggests that nursing students do not engage with civic and community activities uniformly. Previous literature has increasingly emphasized the multidimensional nature of civic engagement, including civic attitudes, civic participation, social responsibility, and community involvement [22]. Our findings extend this evidence by demonstrating that nursing students can be meaningfully categorized into distinct subgroups according to their civic engagement patterns. The largest subgroup in the present study consisted of students with high civic awareness. This suggests that many nursing students possess strong civic values and community-oriented perspectives even when active behavioral participation may vary. This observation is particularly relevant within nursing education, where professional identity formation and commitment to public wellbeing are central components of training.

In our study, the inverse association between civic engagement and psychological distress is one of the core findings. Students belonging to the Attitude Oriented profile demonstrated substantially lower depression, anxiety, and stress scores compared with students in the low-engagement profile. Our findings are consistent with previous evidence suggesting that civic participation and prosocial engagement contribute positively to mental wellbeing [23,24]. Civic engagement may improve mental health through several potential mechanisms, including increased social connectedness, stronger sense of purpose, enhanced self-efficacy, and greater perceived social support. Students who actively engage in community and civic activities may develop broader interpersonal networks and stronger feelings of belonging, both of which are known protective factors against psychological distress.

The association we found between civic engagement and reduced psychological distress may also reflect the psychologically meaningful role of altruism and community contribution [25]. Nursing students who participate in civic and community activities may experience greater personal fulfillment and stronger alignment between personal values and professional identity. Our findings also align with prior studies conducted during and after the COVID-19 pandemic, which found that civic engagement may protect against psychological distress [26]. Another study found that observed that civic participation involving social interaction was associated with lower psychological distress [27].

Interestingly, our study also identified an Attitude Oriented profile characterized by relatively high civic attitudes but lower civic behaviors and community interest. This finding suggests that endorsement of civic values alone may not necessarily translate into active participation. Previous literature has similarly highlighted discrepancies between civic beliefs and actual civic involvement among young adults [22]. Nursing students may particularly experience restricted engagement opportunities because of demanding academic schedules and clinical responsibilities [28]. Our study demonstrates that females showed significantly lower psychological distress scores compared with male students. Although many previous studies have reported higher distress among female university students, contextual and cultural factors may partly explain these findings [29,30,31]. Differences in coping strategies, social support utilization, and engagement in emotionally supportive social networks may contribute to gender related variations in psychological outcomes.

Although the observed associations remained significant after adjustment for several sociodemographic factors, other psychosocial and academic influences may also contribute to the relationship between civic engagement and psychological wellbeing. Social support, resilience, personality characteristics, coping strategies, and academic workload have all been identified as important determinants of mental health among university students [31]. Students with stronger social networks and greater psychological resilience may be more likely to participate in civic and community activities while simultaneously experiencing lower levels of psychological distress. Similarly, adaptive coping strategies may facilitate both engagement in prosocial activities and more effective management of academic and emotional challenges. Therefore, the associations observed in the present study should be interpreted within a broader psychosocial context, and future research should incorporate these variables to better understand the mechanisms linking civic engagement and mental wellbeing.

An interesting observation was that students in the High Civic Awareness profile exhibited the lowest mean levels of depression, anxiety, and stress in the descriptive analyses, whereas the Attitude Oriented profile demonstrated the strongest associations with lower psychological distress after adjustment for demographic and academic characteristics. This apparent difference likely reflects the influence of covariate adjustment, particularly the unequal distribution of gender across the latent profiles, rather than inconsistency between the findings. The multivariable models therefore estimate the independent association between civic engagement profile membership and psychological distress after accounting for potential confounding factors.

### Strengths and Limitations

This study has several strengths. The use of latent profile analysis enabled us to identify the distinct patterns of civic engagement that may not have been captured using conventional variable-centered approaches. By examining combinations of civic attitudes, civic behaviors, and community interest simultaneously, we were able to characterize meaningful subgroups within the student population and demonstrate substantial heterogeneity in engagement patterns. The study also incorporated rigorous psychometric evaluation of the civic engagement scale through exploratory and confirmatory factor analyses, which supports the reliability and construct validity of the instrument within the study population. In addition, the assessment of multiple psychological distress outcomes, including depression, anxiety, and stress, provided a comprehensive evaluation of student mental wellbeing. Finally, our study contributes evidence from nursing students in Saudi Arabia, a population for which research on civic engagement and mental health remains relatively limited.

However, several limitations should be acknowledged. The use of a cross-sectional design in this study may restrict the causal effects of civic engagement on psychological distress. This means that it is possible that students with better psychological wellbeing are more likely to participate in civic activities rather than civic engagement directly improving mental health. The use of self-reported measures may also introduce recall bias, reporting bias, and social desirability bias. Since participants were recruited from a single university, the generalizability of the findings to other nursing student populations and educational settings may be limited. Although latent profile analysis identified meaningful engagement profiles, the identified class structures may vary across different institutional and cultural contexts. Furthermore, although the analyses adjusted for several important sociodemographic variables, residual confounding cannot be excluded. Psychosocial and academic factors such as social support, resilience, personality traits, coping strategies, and academic workload were not measured but may influence both civic engagement and psychological wellbeing. For example, students with stronger social support or greater resilience may be more likely to participate in civic activities while also experiencing lower levels of psychological distress. Similarly, adaptive coping strategies and lower academic workload may facilitate greater civic participation and better mental health. Future longitudinal studies incorporating these factors are needed to clarify the complex pathways linking civic engagement and psychological wellbeing. The study sample consisted predominantly of male students. Although recruitment was open to all eligible students, male and female nursing students are educated on separate campuses within the study institution, and participation was voluntary. As a result, differences in participation rates may have influenced the gender composition of the final sample. Consequently, the findings should be interpreted with caution and may not be fully generalizable to nursing student populations with different gender distributions. Future multicenter studies should seek more balanced representation across campuses and institutions.

## 5. Conclusions

In conclusion, our findings showed that there were distinct civic engagement profiles among nursing students that were significantly associated with psychological distress outcomes. Students with higher levels of civic engagement demonstrated lower depression, anxiety, and stress scores. The findings suggest that promoting civic engagement within nursing education may contribute not only to professional and social development but also to improved psychological health among nursing students. Further longitudinal and multicenter studies are needed to better understand the causal relationship between civic engagement and mental wellbeing in university populations.

## Figures and Tables

**Table 1:** Sociodemographic characteristics, psychological distress, and civic engagement among nursing students (N = 255).

Characteristic	N = 255 n (%)
Age group	
18 to 20 years	74 (29.0)
21 to 23 years	146 (57.3)
24 to 26 years	35 (13.7)
Gender	
Male	172 (67.5)
Female	83 (32.5)
Academic year	
First year	9 (3.5)
Second year	47 (18.4)
Third year	130 (51.0)
Fourth year	46 (18.0)
Internship year	23 (9.0)
Grade point average	
Below 2.5	10 (3.9)
2.5 to 2.99	40 (15.7)
3.0 to 3.49	92 (36.1)
3.5 to 4.0	113 (44.3)
Volunteering frequency	
Never	37 (14.5)
Rarely	73 (28.6)
Occasionally	91 (35.7)
Monthly	33 (12.9)
Weekly	21 (8.2)
Community participation	
No and no plans	32 (12.5)
No but plan to	112 (43.9)
Yes occasionally	78 (30.6)
Yes regularly	33 (12.9)
Psychological distress and civic engagement scores, mean (sd)	
Depression score	12.73 (10.59)
Anxiety score	12.64 (10.87)
Stress score	13.77 (9.81)
Civic engagement score	5.28 (1.20)

**Table 2:** Reliability coefficients and latent profile model fit statistics for civic engagement among nursing students.

Measure/Model	Number of Items	Cronbach’s α	AIC	BIC	SABIC	Entropy	BLRT *p*-Value	Class Distribution (%)
Reliability analysis								
Civic engagement scale	14	0.93	-	-	-	-	-	-
Depression subscale	7	0.90	-	-	-	-	-	-
Anxiety subscale	7	0.90	-	-	-	-	-	-
Stress subscale	7	0.86	-	-	-	-	-	-
Latent profile analysis								
1-class solution	-	-	2179.97	2201.22	2182.20	1.000	-	-
2-class solution	-	-	1927.70	1963.11	1931.41	0.834	<0.01	49.4/50.6
3-class solution	-	-	1892.85	1942.43	1898.04	0.812	<0.01	22.4/46.3/31.3
4-class solution	-	-	1831.08	1894.82	1837.76	0.805	<0.01	16.9/22.0/43.1/18.0
5-class solution	-	-	1791.01	1868.92	1799.17	0.846	<0.01	20.4/19.6/41.2/4.7/14.1

**Table 3:** Sociodemographic characteristics and psychological distress across latent civic engagement profiles among nursing students. Values are presented as n (%) or mean (SD). *p*-values were obtained using chi-squared test for categorical variables and one-way ANOVA for continuous variables. For variables with sparse cell counts (academic year and GPA), chi-square tests with Monte Carlo simulated *p*-values (10,000 replicates) were used.

Characteristic	Low Engagement (N = 43)	Moderate Engagement (N = 56)	High Civic Awareness (N = 110)	Attitude Oriented (N = 46)	*p*-Value
Age group					0.060
18 to 20 years	15 (34.9)	15 (26.8)	26 (23.6)	18 (39.1)	
21 to 23 years	23 (53.5)	37 (66.1)	61 (55.5)	25 (54.3)	
24 to 26 years	5 (11.6)	4 (7.1)	23 (20.9)	3 (6.5)	
Gender					<0.001
Male	43 (100.0)	48 (85.7)	36 (32.7)	45 (97.8)	
Female	0 (0.0)	8 (14.3)	74 (67.3)	1 (2.2)	
Academic year					0.009
First year	1 (2.3)	0 (0.0)	7 (6.4)	1 (2.2)	
Second year	13 (30.2)	10 (17.9)	15 (13.6)	9 (19.6)	
Third year	20 (46.5)	35 (62.5)	47 (42.7)	28 (60.9)	
Fourth year	7 (16.3)	9 (16.1)	23 (20.9)	7 (15.2)	
Internship year	2 (4.7)	2 (3.6)	18 (16.4)	1 (2.2)	
Grade point average					0.004
Below 2.5	5 (11.6)	3 (5.4)	1 (0.9)	1 (2.2)	
2.5 to 2.99	11 (25.6)	6 (10.7)	18 (16.4)	5 (10.9)	
3.0 to 3.49	8 (18.6)	26 (46.4)	45 (40.9)	13 (28.3)	
3.5 to 4.0	19 (44.2)	21 (37.5)	46 (41.8)	27 (58.7)	
Volunteering frequency					0.043
Never	8 (18.6)	9 (16.1)	8 (7.3)	12 (26.1)	
Rarely	8 (18.6)	17 (30.4)	30 (27.3)	18 (39.1)	
Occasionally	15 (34.9)	20 (35.7)	47 (42.7)	9 (19.6)	
Monthly	8 (18.6)	4 (7.1)	17 (15.5)	4 (8.7)	
Weekly	4 (9.3)	6 (10.7)	8 (7.3)	3 (6.5)	
Psychological distress scores					
Depression score	17.67 (10.14)	17.25 (9.59)	9.07 (9.66)	11.35 (10.73)	<0.001
Anxiety score	17.91 (10.53)	16.61 (9.54)	8.24 (10.19)	13.43 (10.33)	<0.001
Stress score	18.33 (8.60)	17.46 (8.58)	10.89 (9.39)	11.91 (10.50)	<0.001

**Table 4:** Multivariable linear regression analysis examining the association between civic engagement profiles and psychological distress outcomes among nursing students. The reference categories were Low Engagement profile, age 18 to 20 years, male gender, first academic year, and the less than 2.5 GPA category.

Variable	Depression β (95% CI)	Anxiety β (95% CI)	Stress β (95% CI)
Civic engagement profiles			
Moderate Engagement	0.55 (−3.46, 4.56)	0.00 (−4.00, 4.00)	−0.03 (−3.76, 3.70)
High Civic Awareness	−4.85 (−9.13, −0.57)	−4.20 (−8.47, 0.07)	−3.45 (−7.43, 0.53)
Attitude Oriented	−6.63 (−10.79, −2.48)	−4.72 (−8.87, −0.58)	−6.95 (−10.81, −3.09)
Age group			
21 to 23 years	−4.11 (−7.49, −0.74)	−3.76 (−7.12, −0.39)	−3.39 (−6.53, −0.25)
24 to 26 years	4.46 (−2.06, 10.99)	5.14 (−1.37, 11.65)	1.97 (−4.10, 8.03)
Gender			
Female	−5.75 (−9.61, −1.89)	−7.61 (−11.46, −3.76)	−6.00 (−9.59, −2.41)
Academic year			
Second year	0.01 (−7.23, 7.26)	1.09 (−6.15, 8.32)	0.54 (−6.20, 7.28)
Third year	2.31 (−5.08, 9.71)	3.82 (−3.55, 11.20)	3.42 (−3.46, 10.30)
Fourth year	3.10 (−4.93, 11.14)	3.06 (−4.96, 11.07)	1.33 (−6.14, 8.81)
Internship year	−4.96 (−15.04, 5.12)	−5.47 (−15.53, 4.59)	−2.11 (−11.49, 7.26)
Grade point average			
2.5 to 2.99	2.16 (−2.39, 6.71)	1.23 (−3.31, 5.77)	3.09 (−1.14, 7.32)
3.0 to 3.49	−1.72 (−5.53, 2.10)	−0.29 (−4.09, 3.52)	−2.44 (−5.99, 1.11)
3.5 to 4.0	0.94 (−1.88, 3.77)	0.67 (−2.16, 3.49)	1.94 (−0.69, 4.57)

## Data Availability

The raw data supporting the conclusions of this article will be made available by the authors on request.

## Supplementary Materials

The following supporting information can be downloaded at: https://www.mdpi.com/article/10.3390/healthcare14142155/s1, Figure S1: Pearson correlation matrix of civic engagement and psychological distress domains among nursing students; Figure S2: Diagnostic plots for the multivariable linear regression models. Residual diagnostic plots for the depression, anxiety, and stress regression models. For each model, the four standard diagnostic plots are presented: residuals versus fitted values, normal Q-Q plot, scale-location plot, and residuals versus leverage plot; Table S1: Exploratory factor analysis of the civic engagement scale among nursing students; Table S2: Confirmatory factor analysis of the civic engagement scale among nursing students.
