# Supplementary material for: Profiles of Civic Engagement and Their Association with Depression, Anxiety, and Stress Among Nursing Students in Saudi Arabia
Source: Healthcare (Basel). 2026 Jul 17;14(14):2155. doi: 10.3390/healthcare14142155 (PMC13411811; doi:10.3390/healthcare14142155)
Supplement: Supplementary file 1 [file healthcare-14-02155-s001.zip › healthcare-4374711-supplementary.pdf]

Supplementary Materials

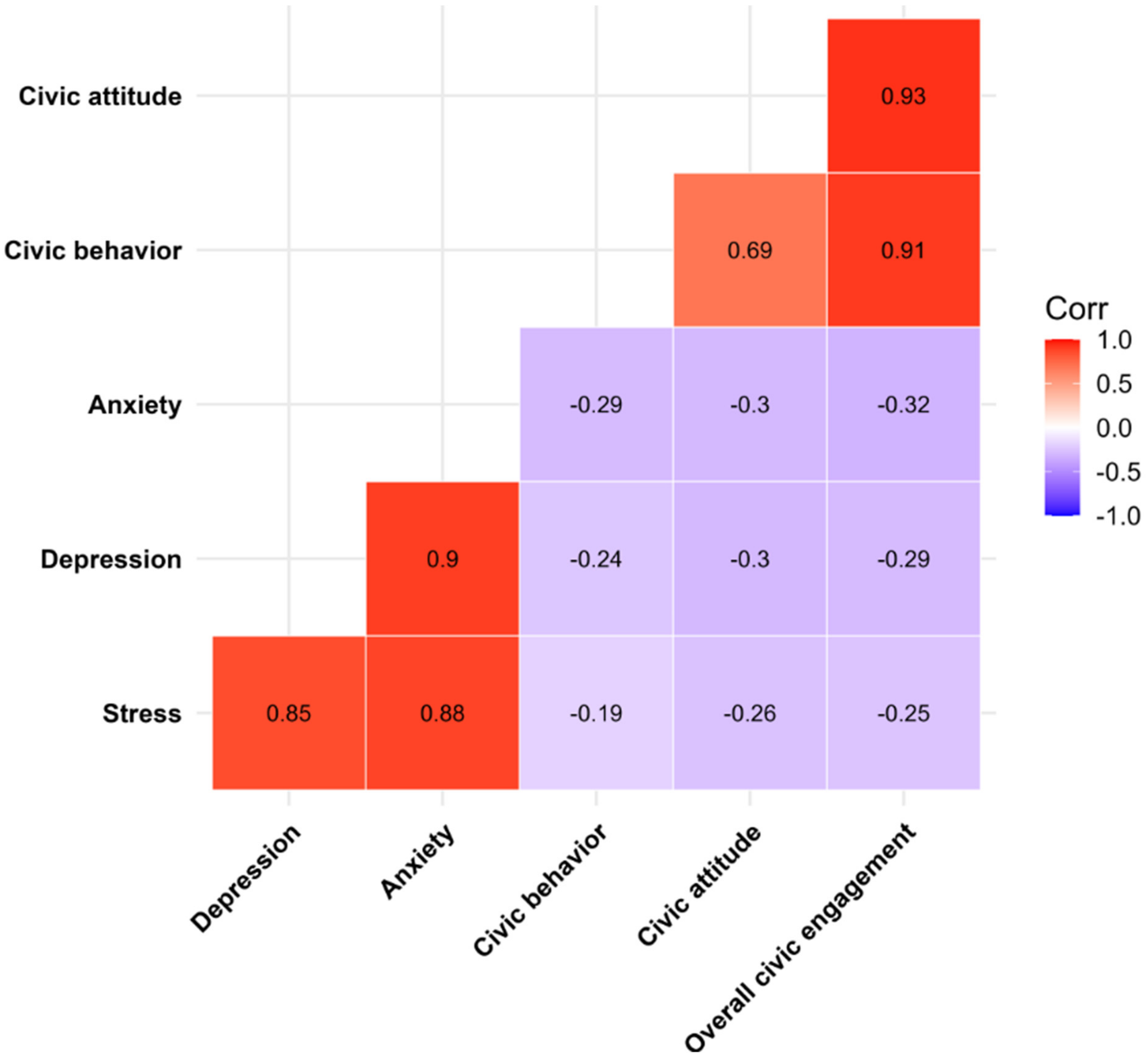

Figure S1: Pearson correlation matrix of civic engagement and psychological distress domains among nursing students.

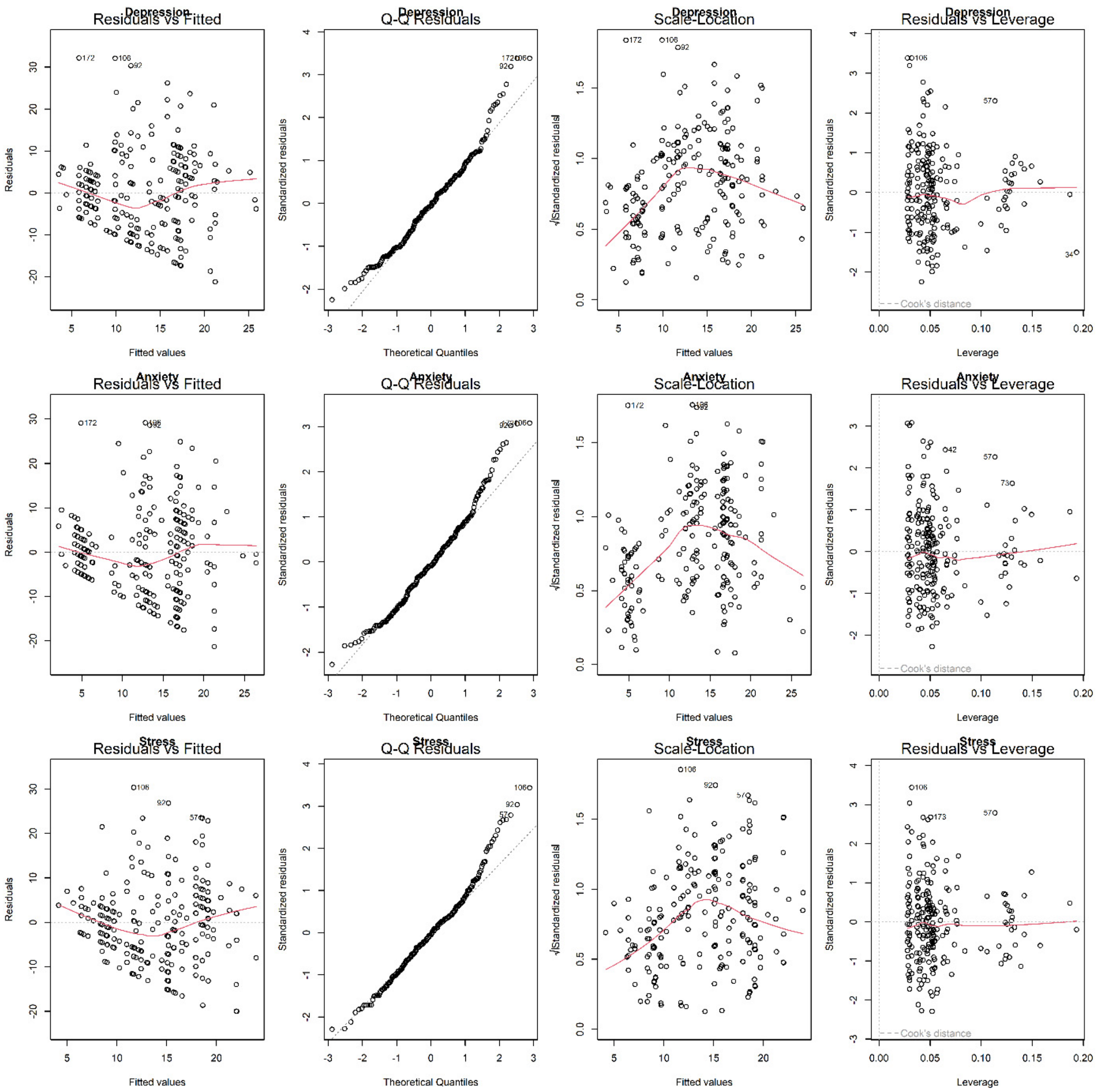

Figure S2: Diagnostic plots for the multivariable linear regression models. Residual diagnostic plots for the depression, anxiety, and stress regression models. For each model, the four standard diagnostic plots are presented: residuals versus fitted values, normal Q-Q plot, scale-location plot, and residuals versus leverage plot.

Table S1. Exploratory factor analysis of the civic engagement scale among nursing students.

Item	Factor 1: Civic Attitude	Factor 2: Civic Behavior	Communality (h^2)
a1	0.76	—	0.59
a2	0.69	—	0.56
a3	0.85	—	0.68
a4	0.82	—	0.68
a5	0.81	—	0.57
a6	0.73	—	0.55
a7	0.53	—	0.51
a8	0.57	—	0.48
b1	—	0.81	0.54
b2	—	0.62	0.63
b3	0.38	0.48	0.62
b4	—	0.67	0.65
b5	—	0.82	0.62
b6	—	0.78	0.62

Table S2. Confirmatory factor analysis of the civic engagement scale among nursing students.

**Item / Fit Statistic**	**Civic Attitude**	**Civic Behavior**
**a1**	0.764	—
**a2**	0.753	—
**a3**	0.820	—
**a4**	0.809	—
**a5**	0.747	—
**a6**	0.746	—
**a7**	0.715	—
**a8**	0.699	—
**b1**	—	0.685
**b2**	—	0.813
**b3**	—	0.800

**b4**	—	0.816
**b5**	—	0.739
**b6**	—	0.748
**Model fit statistics**	**Value**	
**CFI**	0.921	
**TLI**	0.906	
**RMSEA**	0.096	
**RMSEA 90% CI**	0.083–0.109	
**SRMR**	0.052	
